# Effects of Puerarin on Lipid Accumulation and Metabolism in High-Fat Diet-Fed Mice

**DOI:** 10.1371/journal.pone.0122925

**Published:** 2015-03-30

**Authors:** Guodong Zheng, Lezhen Lin, Shusheng Zhong, Qingfeng Zhang, Dongming Li

**Affiliations:** 1 Jiangxi Key Laboratory of Natural Product and Functional Food, College of Food Science and Engineering, Jiangxi Agricultural University, Nanchang, China; 2 Library of Jiangxi Agricultural University, Nanchang, China; Monash University, AUSTRALIA

## Abstract

In order to investigate the mechanisms by which puerarin from kudzu root extract regulates lipid metabolism, fifty mice were randomly assigned to five groups: normal diet, high-fat diet (HFD), and HFD containing 0.2%, 0.4% or 0.8% puerarin for 12 weeks. Body weight, intraperitioneal adipose tissue (IPAT) weight, serum biochemical parameters, and hepatic and feces lipids were measured. Activity and mRNA and protein expressions of hepatic lipid metabolism-related enzymes were analyzed. Compared with HFD, 0.4% and 0.8% puerarin significantly decreased body and IPAT weight. There was a significant decrease in the serum and hepatic concentrations of total cholesterol, triglycerides and leptin in mice fed the 0.4% and 0.8% puerarin diets compared with HFD. Fatty acid synthase activity was suppressed in mice fed the 0.4% and 0.8% puerarin diets, while the activities of AMP-activated protein kinase (AMPK), carnitine acyltransferase (CAT) and hormone-sensitive lipase (HSL) were increased. mRNA expression of *peroxisome proliferator-activated receptor γ 2 (PPARγ 2)* was down-regulated in liver of mice fed the 0.8% diet compared with HFD, while mRNA expression of *CAT* and *HSL* was considerably up-regulated by 0.4% and 0.8% puerarin diets. The protein expression of PPARγ2 in liver was decreased and those of p-AMPK, HSL and p-HSL were increased in mice fed 0.4% and 0.8% puerarin diets. These results suggest that > 0.4% puerarin influenced the activity, mRNA and protein levels of hepatic lipid metabolism-related enzymes, decreasing serum and liver lipids, body weight gain and fat accumulation. Puerarin might be beneficial to prevent lifestyle-related diseases.

## Introduction

Obesity has become a public health concern since it is associated with type 2 diabetes mellitus, hypertension, stroke, dyslipidemia, cardiovascular disease, osteoarthritis and some cancers [1]. Therefore, prevention of obesity plays a role in the prevention of these diseases. Excessive macronutrient intake contributes to obesity [2], whereas some dietary polyphenols and flavonoids are able to reduce the incidence of obesity [3,4]. Many compounds in plants are now recognized to be useful for human health, but safety assessment and interactions of these botanical compounds need to be further elucidated.

Puerarin (7, 4’-dihydroxyisolavone-8-β-glucopyranoside) is an active isoflavone extracted from the roots of Pueraria lobata (Willd.) Ohwi. Puerarin is widely used in traditional Chinese medicine, and is clinically used in China for the treatment of coronary artery disease, heart failure, hypertension and myocardial infarction [5]. Pharmacokinetic parameters of puerarin were determined by oral administration (400 mg/kg) of puerarin to rats; serum levels peaked after 25 minutes at 18.66 mg/L, and t_1/2_ was about 4h [6]. It has been reported that puerarin had therapeutic effects on hypertension, diabetes mellitus, arteriosclerosis and myocardial ischemia in rats [7,8]. Puerarin attenuates angiotensin II-induced cardiac hypertrophy in mice [9], and alleviates neuropathic pain in rat models of chronic constriction injury and diabetic neuropathy [10]. Puerarin is a substrate for cytochrome P450 (CYP450) and P-glycoprotein, which are critical functional proteins in drug metabolism and transport. Therefore, concomitant administration of drugs that influence CYP450 and/or P-glycoprotein should change the pharmacokinetics of puerarin [11,12]. Wang et al. [13] reported that puerarin facilitates t-tubule development of murine embryonic stem cell-derived cardiomyocytes. Puerarin has hepatoprotective effects in chronic alcohol-induced injury in rats [14] mediated, at least in part, by decreased endotoxin receptor expression in rats [15] and by the regulation of the GSK-3β/NF-κB pathway [16]. Puerarin can inhibit plasma renin activity in spontaneously hypertensive rats [17]. Therefore, puerarin has attracted considerable attention because of its putative ability to protect against metabolic disorders [18].

However, the effects of puerarin on lipid metabolism are not clear. In the present study, we aimed to investigate the anti-obesity effects of puerarin through its effects on body weight, intraperitioneal adipose tissue (IPAT), serum biochemical parameters, as well as on activity, mRNA and protein levels of hepatic lipid metabolism enzymes in mice.

## Materials and Methods

### Ethics statement

This study was carried out in strict accordance with the recommendations from the Guide for the Care and Use of Laboratory Animals of the Chinese Association for Laboratory Animal Science. All animal care and protocols were approved by the Animal Care and Use Committee of the Jiangxi Agricultural University. All sacrifices were performed under sodium pentobarbital anesthesia, and efforts were taken to minimize animal suffering.

### Animals and diets

Four-week-old female ICR mice, weighing about 20 g, were purchased from Hunan Silaike Laboratory Animal Co., Ltd (Changsha, China). Puerarin (>90%) was obtained from Nanjing Jing Zhu Biological Technology Co., Ltd (Nanjing, China).

All mice were acclimated on a normal diet for one week. Then, fifty mice were divided into five groups: normal diet (controls), high-fat diet (HFD), and HFD supplemented with 0.2%, 0.4% or 0.8% puerarin, which the doses were equivalent to content of 2.5%, 5% or 10% kudzu root powder diets. The compositions of the diets are shown in S1 Table. Mice were allowed free access to food and tap water for 12 weeks. During the feeding period, the mice were weighed every week. Mice were anesthetized and sacrificed after the experiment, and blood was sampled from the heart. Serum was isolated by centrifugation at 825 g for 15 min at 4°C. Serum was aliquoted and immediately stored at -80°C. The liver and IPAT of each mouse were harvested and weighed. Livers (and IPAT tissues) were cut into five pieces, washed with saline, and frozen at -80°C. Food intake was measured every day in twelfth week. Feces were collected every day for two weeks and were dried at 60°C in a thermostatic oven after collection. All mice were housed (5 mice/cage) in an air-conditioned (temperature 24 ± 2°C, humidity 50 ± 10%) and light-controlled (12:12 h light:dark cycle, light from 08:00 to 20:00 hours) animal room.

### Biochemical analysis in the serum, liver and feces

Serum levels of triglycerides (TG), total cholesterol (TC) and glucose were analyzed using commercial kits (Biosino Biotechnology and Science Inc., Beijing, China), according to the manufacturer's instructions. Non-esterified fatty acids (NEFA) were determined using a commercial kit (Nanjing Jiancheng Bioengineering Institute, Jiangsu, China), according to the manufacturer's instructions. Serum leptin and insulin concentrations were measured using ELISA kits (R&D systems, Minnesota, USA). Total lipid were extracted from the liver and feces according to the method of Folch *et al* [19]. TG and TC contents of the liver and feces were analyzed using commercial kits (Biosino Biotechnology and Science Inc, Beijing, China).

### Hepatic lipid metabolism-related enzymes activities

The frozen liver was homogenized in buffer A (3 mmol/L Tris/HCl, pH 7.2, 1 mmol/L EDTA, 1 mmol/L dithiothreitol, 25 μmol/L ALLN (a calpain and cathepsin inhibitor, N-acetyl-leucyl-leucyl-norl-leucina), 100 μmol/L leupeptin, 100 μmol/L AEBSF (a serine protease inhibitor,4-(2-aminoethy1) bezenesulfonyl-fluoride), 10 μmol/L E64, and 0.25 mol/L sucrose) according to the method by Zheng *et al*. [20]. Protein concentration of the homogenate was measured using a kit based on the Coomassie brilliant blue method (#A045-2; NanJing JianCheng Bioengineering Institute, NanJing, China) and adjusted to 10 mg/ml for carnitine acyltransferase (CAT) activity analysis. The homogenate was centrifuged at 500 g for 10 min and the supernatant was used in assays for acyl-CoA oxidase (ACO) activity. The supernatant (500 g) was further centrifuged at 9000 g for 15 min and this supernatant was used for the analysis of acetyl-CoA carboxylase (ACC), fatty acid synthase (FAS), AMP-activated protein kinase (AMPK) and hormone-sensitive lipase (HSL) activities. The activities of ACC, FAS, AMPK, ACO, CAT and HSL in the liver were analyzed by commercial kits. The measurement principle of FAS is that fatty acids are synthesized from acetyl coenzyme A and malonyl coenzyme A by FAS with NADPH as the hydrogen donor. The enzymatic activity of FAS is directly positively correlated to the decrease rate of NADPH, which is used for quantification of FAS activity. Five μL of liver homogenate supernatant were added into each well of a 96-well plate; 76 μL of potassium phosphate buffer solution (0.2 mmol/L, pH 7.0) was added, followed 19 μL of a mixture containing acetyl coenzyme A (150 μmol/L), NADPH (2 mmol/L), EDTA (1 mmol/L), DTT (5 mmol/L), and malonyl coenzyme A (0.5 mmol/L). The reaction was measured at 340 nm and 37°C. The measurement principle of CAT is that in the presence of carnitine, coenzyme A is hydrolyzed from acyl coenzyme A by acyl carnitine transferase, and coenzyme A reacts with 5,5-dithiobis-2-nitrobenzoic acid (DTNB) to generate a yellow-colored product (TNB) that is positively correlated to CAT enzymatic activity and therefore used for quantitative measurement of CAT. Five μL of liver homogenate supernatant were added into each well of a 96-well plate with 95 μL of Tris-HCl buffer solution (pH 8.0, containing 0.5 mmol/L EDTA, 0.3 mmol/L DTNB, 0.1% Triton X-100, 1.5 mmol/L carnitine, and 50 μmol/L stearoyl coenzyme A). The reaction was measured at 412 nm and 37°C. The measurement principle of ACO is that the β-oxidation of fatty acids generates unsaturated fatty acids, and that hydrogen peroxide is further produced by oxidation. Hydrogen peroxide reacts with polyphenols, aminopyrine, and peroxidase to produce red colored products that are used for quantitative analysis. Five μL of liver homogenate supernatant were added into each well of a 96-well plate with 90 μL of potassium phosphate buffer solution (pH 7.4, containing 1.64 mmol/L aminopyrine, 15 mmol/L, 10 mmol/L, 4 units FAD polyphenol peroxidase, and 0.1 mmol/L bovine albumin), followed by 5 μL of stearoyl coenzyme A. The reaction was measured at 500 nm and 37°C. The measurement principle of ACC is used purified mouse ACC to coat microtiter plate wells, make solid-phase antibody, then add ACC to wells, Combined ACC which with horseradish peroxidase (HRP) labeled, become antibody-antigen-enzyme complex, then add TMB, TMB substrate becomes blue color. The reaction is terminated by the addition of a sulphuric acid solution and the color change that are used for analysis. Ten μL of liver homogenate supernatant were added into each well of a 96-well plate with 40 μL of sample dilution buffer solution, incubate 30 min at 37°C. Discard on the liquid, with buffer cleaning 3 times, add 50 μL HRP-conjugate reagent to each well, incubate 30 min at 37°C. Add 50 μL chromogen solution A and 50 μL chromogen solution B and incubate at 37°C for 15 min during coloring. Then add 50 μL stop solution to terminate the reaction. The determination of wavelength is at 450 nm. The measurement principle and method of HSL and AMPK refer to ACC.

### mRNA expression of hepatic lipid metabolism-related enzymes

Total RNA was isolated from frozen livers using Trizol (Invitrogen), according to the manufacturer’s instructions. RNA (1 μg) was reverse-transcribed using the cDNA Reverse Transcription Kit (Takara Bio Inc, Japan), according to the manufacturer’s protocols. Real-time quantitative PCR (RT-qPCR) was performed using an Applied Biosystems 7900HT Real-Time PCR System (Applied Biosystems, USA) and Premix Ex Taq (Probe qPCR), according to the protocols provided by the manufacturer. Briefly, PCR was performed in a final volume of 20 μL containing 800 ng of cDNA, 0.4 μL of 0.4 mmol/L forward and reverse primers, 0.4 μL of 0.4 mmol/L fluorescence probe, 10 μL of 1.25 U/25μL Premix Ex Taq, and 0.4 μL of 25 μmol/L ROX Reference Dye. PCR reactions consisted of an initial denaturing step at 95°C for 30 s, followed by 45 cycles of 10 s at 94°C and 37 s at 60°C. Primers and probes are presented in Table 1. Results are presented as levels of expression relative to those of controls after normalization to *β-actin* and *glyceraldehyde 3-phosphate dehydrogenase (GADPH)* using the 2^-ΔΔCT^ method.

**Table 1:** Gene-specific primers and probes used in quantitative real-time PCR.

Gene	Forward	Reverse	Probe	gene accession number
FAS	5’-GGGCTCTATGGATTACC-3’	5’-CATAGCTGACTTCCAACA-3’	5’-CCAAGCAGGCACACACAATGGACC-3’	NM_007988.3
AMPK	5’-TGAAGATCGGCC ACTACATCCT-3’	5’-CTTGCCCACCTTCACTTTCC-3’	5’-ACACGCTTGGTGTCGGCACCTTC-3’	NM_001013367.3
Pparg	5'-ATGATGGGAGAAGATAAAATCAAGTTC-3'	5'-GGATGGCCACCTCTTTGCT-3'	5'-AACATATCACCCCCCTGCAGGAGCA-3'	NM_001127330.1
CAT	5’-CTGTGGGATGGTGTATGAGCAT-3’	5’-GACATGGTCCACAAGTGCAACT-3’	5’-CAGCTGCAG AAGGGCCCCCC-3’	NM_007760.3
ACO	5’- TCACAGCAGTGGGATTCCAA -3’	5’-TCTGCAGCATCATAACAGTGTTCTC-3’	5’-TATTTACGTCACGTTTACCCCGGCCTG-3’	NM_015729.3
HSL	5’-GGAGCACTACAAACGCAACGA-3’	5’-TCGGCCACCGGTAAAGAG-3’	5’-ACAGGCCTCAGTGTGACCGCCA-3’	NM_001039507.2
β-actin	5’ AGAAAATCTGGCACCACACC 3’	5’ CCATCTCTTGCTCGAAGTCC 3’	5′-TAAAACGCAGCTCAGTAACAGTCG-3′	NM_000079.6
GADPH	5’-TGTGTCCGTCGTGGATCTGA-3’	5’-CCTGCTTCACCACCTTCTTGA-3’	5'-TGCCGCCTGGAGAAACCTGCC-3'	NM_008084.2

FAS: fatty acid synthase; AMPK: AMP-activated protein kinase; PPARγ2: peroxisomes proliferator-activated receptor γ2; CAT: carnitine acyltransferase; ACO: acyl-CoA oxidase; HSL: hormone-sensitive lipase; GADPH: glyceraldehyde 3-phosphate dehydrogenase.

### AMPK, P-AMPK, PPARγ, HSL and p-HSL protein expression in the liver

Frozen livers were ground in liquid nitrogen and lysed in RIPA buffer (50 mmol/L Tris, pH 7.4, 150 mmol/L NaCl, 1% Triton X-100, 0.5% sodium deoxycholate, 0.1% SDS, 1 mmol/L EDTA, 1 mmol/L PMSF and 2 μg/ml leupeptin) at 4°C for 1 h. Liver lysates were centrifuged at 9000 g for 15 min, the supernatant was used for measuring the protein expression of AMPK, phosphorylation AMPK (p-AMPK), peroxisomes proliferator-activated receptor γ2 (PPARγ2), HSL, and phosphorylation HSL (p-HSL). Protein concentration of the homogenate was measured using a kit based on the Coomassie brilliant blue method (#A045-2; NanJing JianCheng Bioengineering Institute, NanJing, China). Equal amounts of protein were separated by 8% SDS-PAGE (Beyotime Institute of Biotechnology, Haimen, China) and transferred onto polyvinylidene diflyoride membranes (Millipore, MA, USA). The AMPK, p-AMPK, PPARγ, HSL and p-HSL blots were blocked with 5% non-fat dry milk-TBST buffer (TBS containing 0.1% Tween-20) for 2 h at room temperature. The membranes were rinsed three times for 10 min each with TBST buffer, and then incubated overnight at 4°C with 1:1000 dilutions of antibodies for AMPK (#2793, Cell Signaling Technology Inc. MA, USA), p-AMPK (Thr172) (#2535, Cell Signaling Technology Inc. MA, USA), PPARγ (#2430, Cell Signaling Technology Inc. MA, USA), HSL (#4107, Cell Signaling Technology Inc. MA, USA), or p-HSL (Ser660) (#4126, Cell Signaling Technology Inc. MA, USA). Equal lane loading was assessed using β-actin (Zhongshan Bio Co. Ltd., Beijing, China). The blots were rinsed three times with TBST buffer for 10 min each. Washed blots were incubated with a 1:1000 dilution of a horseradish peroxidase conjugated-secondary antibody (ZSGB-Bio., Beijing, China) for 2h and washed three times with TBST buffer. The transferred proteins were visualized with an enhanced 3,3’-diaminobenzidine tetrahydrochloride kit (ZSGB-Bio., Beijing, China).

### Statistical analysis

All data are presented as means ± standard error of the mean (SEM). Continuous variables were tested for normality, and then analyzed using one-way ANOVA with the Tukey post hoc analysis. Statistical analysis was performed with SPSS 17.0 (SPSS Inc., Chicago, USA). Two-sided P-values of less than 0.05 were considered to be significant.

## Results

### Body weight, organ weight and food intake

The effects of puerarin on body weight, liver weight, IPAT weight and food intake of mice are presented in Table 2. Body weight gain was significantly decreased in mice fed 0.4% and 0.8% puerarin diets compared with HFD. Moreover, IPAT weight was remarkably reduced by 0.4% and 0.8% puerarin compared with HFD. Results indicate that >0.4% puerarin decreased body weight gain and IPAT weight of mice fed HFD.

**Table 2:** Effects of puerarin on body weight, liver weight and IPAT weight and energy intake in mice.

	Initial body weight (g)	Final body weight (g)	Body weight gain (g)	Liver (g/100g bw)	IPAT (g/100g bw)	Energy intake (kcal/d)
Control	23.23±0.40	35.51±1.07^c^	12.29±0.89^c^	4.36±0.13^ab^	3.15±0.12^d^	19.47±0.68^b^
HFD	23.31±0.39	42.93±1.27^a^	19.62±1.01^a^	3.85±0.17^b^	7.91±0.33^a^	22.52±0.95^a^
HFD + 0.2% puerarin	23.34±0.42	40.09±1.03^ab^	16.75±0.95^ab^	4.06±0.30^ab^	7.03±0.28^a^	22.19±1.05^ab^
HFD + 0.4% puerarin	22.98±0.38	37.57±0.97^bc^	14.59±0.90^bc^	4.31±0.15^ab^	5.07±0.16^b^	22.77±0.90^a^
HFD + 0.8% puerarin	23.10±0.37	36.63±0.90^c^	13.52±0.76^c^	4.49±0.19^a^	3.91±0.14^c^	22.62±0.89^a^

Values are means ± SEM of 10 mice. Means within the same column but not sharing the same superscript letter are significantly different (P<0.05).

Energy from sucrose, com starch, dextrin and casein were 4 kcal/g. Energy from corn oil and beef tallow were 9 kcal/g. Energy from cellulose was 0 kcal/g.

### Serum biochemical parameters, hepatic and feces lipid profiles

Table 3 shows the serum biochemical parameters and hepatic and feces lipid profiles in mice. Serum TC and leptin concentrations were remarkably lowered in mice fed 0.4% and 0.8% puerarin compared with the HFD. There was a significant decrease in serum and hepatic TG levels of mice fed the 0.8% puerarin diet compared with the HFD group.

**Table 3:** Effect of puerarin on serum biochemical parameters and hepatic and feces lipid levels in mice.

	Control	HFD	HFD + 0.2% puerarin	HFD + 0.4% puerarin	HFD + 0.8% puerarin
Serum biochemical parameters					
GLU (mmol/L)	3.38±058	3.50±0.53	3.45±0.48	3.23±0.69	3.49±0.61
TC (mmol/L)	2.31±0.10^c^	3.48±0.17^a^	2.89±0.17^ab^	2.47±0.16^bc^	2.15±0.17^c^
TG (mmol/L)	1.37±0.14^c^	2.54±0.17^a^	1.71±0.11^bc^	2.17±0.20^ab^	1.44±0.17^bc^
NEFA (μg/L)	3.55±0.33	3.73±0.45	3.65±0.44	3.56±0.30	3.88±0.43
Leptin (ng/L)	570.4±17.2^c^	933.4±21.7^a^	795.0±22.9^ab^	742.4±17.3^b^	605.3±12.3^c^
Insulin (mU/L)	10.03±0.14a	10.32±0.11	10.05±0.14	9.92±0.15	9.83±0.13
Liver lipids (μmol/g liver weight)					
TC	14.29±0.99^c^	21.92±1.59^a^	19.00±1.73^ab^	14.76±1.36^c^	17.02±1.07^bc^
TG	27.11±3.74^b^	40.92±4.97^a^	33.33±4.76^ab^	34.56±3.78^ab^	30.12±4.15^b^
Feces lipids (mg/g dry feces)					
TC	4.23±0.33	4.72±0.42	4.54±0.43	4.38±0.45	4.21±0.39
TG	7.84±0.73^b^	9.60±0.88^ab^	10.42±0.92^ab^	10.79±1.03^ab^	11.91±0.95^a^

TC: total cholesterols; TG: triglycerides; GLU: glucose; NEFA: non-esterified fatty acids.

Values are means ± SEM of 10 mice. Means within the same row but not sharing the same superscript letter are significantly different (*P*<0.05).

### Activities of hepatic lipid metabolism-related enzymes

The activities of ACC, FAS, AMPK, CAT, ACO and HSL in mice livers are shown in Table 4. The activity of FAS was significantly decreased in mice fed the 0.4% and 0.8% puerarin diets compared with the HFD group. CAT activity was markedly increased by 0.8% puerarin compared with the HFD group. HSL and AMPK activities were significantly increased in mice fed 0.4% and 0.8% puerarin diets as compared with the HFD group.

**Table 4:** Effect of puerarin on activities of hepatic lipid metabolism-related enzymes (U/g of liver).

	ACCα	FAS	AMPK	CAT	ACO	HSL
Control	7.53±0.41	1.75±0.10^a^	1.54±0.06^c^	1.53±0.14^c^	1.88±0.11^b^	0.85±0.08^c^
HFD	6.86±0.35	1.64±0.08^a^	1.48±0.08^c^	2.32±0.09^b^	2.56±0.12^a^	0.92±0.08^c^
HFD+0.2% puerarin	6.94±0.39	1.28±0.08^ab^	1.74±0.09^bc^	2.43±0.13^ab^	2.01±0.15^ab^	0.99±0.10^bc^
HFD+0.4% puerarin	6.80±0.32	0.98±0.06^b^	1.96±0.06^ab^	2.56±0.07^ab^	2.65±0.16^a^	1.21±0.07^ab^
HFD+0.8% puerarin	7.01±0.48	1.02±0.06^b^	2.15±0.09^a^	2.87±0.09^a^	2.69±0.15^a^	1.37±0.09^a^

ACCα: acetyl-CoA carboxylase α; FAS: fatty acid synthase; AMPK: AMP-activated protein kinase; CAT: carnitine acyltransferase; ACO: acyl-CoA oxidase; HSL: hormone-sensitive lipase.

Values are means ± SEM of 10 mice. Means within the same column but not sharing the same superscript letter are significantly different (*P*<0.05).

### mRNA expression of hepatic lipid metabolism-related enzymes

mRNA expression levels of hepatic lipid metabolism-related enzymes were analyzed by RT- qPCR, and the results are presented in Table 5. mRNA expression levels of PPARγ2 was significantly down-regulated in mice fed 0.4% and 0.8% puerarin diets compared with the HFD group. Compared with the HFD group, there was a significant up-regulation of mRNA expression levels of *CAT* and *HSL* by 0.4% and 0.8% puerarin, and that of *ACO* by 0.4% puerarin.

**Table 5:** Effect of puerarin on mRNA expression of hepatic lipid metabolism-related enzymes.

	*FAS*	*AMPK*	*PPARγ2*	*CAT*	*ACO*	*HSL*
Control	1.00±0.08	1.00±0.06	1.00±0.07^a^	1.00±0.06^b^	1.00±0.09^c^	1.00±0.09^cd^
HFD	0.91±0.08	1.02±0.06	0.95±0.07^a^	1.03±0.07^b^	1.07±0.11^c^	1.16±0.09^bc^
HFD + 0.2% puerarin	0.92±0.07	1.00±0.07	0.90±0.08^ab^	1.10±0.08^ab^	1.20±0.10b^c^	1.52±0.13^ab^
HFD + 0.4% puerarin	0.93±0.09	1.05±0.06	0.72±0.06^bc^	1.32±0.08^a^	1.68±0.11^a^	1.96±0.13^a^
HFD + 0.8% puerarin	0.91±0.10	1.09±0.08	0.56±0.05^c^	1.31±0.07^a^	1.57±0.10^ab^	2.05±0.16^a^

The housekeeping genes used *β-actin* and *GADPH*.

*FAS: fatty acid synthase; AMPK: AMP-activated protein kinase; PPARγ2: peroxisomes proliferator-activated receptor γ2; CAT: carnitine acyltransferase; ACO: acyl-CoA oxidase; HSL: hormone-sensitive lipase*.

Values are means ± SEM of 10 mice. Means within the same column but not sharing the same superscript letter are significantly different (*P*<0.05).

### Protein expressions of FAS, PPARγ2, HSL and p-HSL in the liver

**Fig 1:**
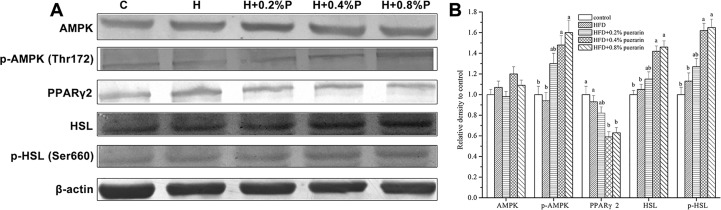
Effects of puerarin on the hepatic protein expression levels of AMPK, p-AMPK, PPARγ2, HSL and p-HSL. A: Western blot analysis of AMPK, p-AMPK, PPARγ2, HSL and p-HSL from the mice liver. B: Expression of AMPK, p-AMPK, PPARγ2, HSL and p-HSL protein normalized to β-actin and expressed relatively to control. Values are means ± SEM of 10 mice. Means within the same column but not sharing the same superscript letter are significantly different (P<0.05). AMPK: AMP-activated protein kinase; p-AMPK: phosphorylation of AMP-activated protein kinase; PPARγ2: peroxisomes proliferator-activated receptor γ2; HSL: hormone-sensitive lipase; p-HSL: phosphorylation of hormone-sensitive lipase.

Fig 1 presents the protein expressions of AMPK, p-AMPK, PPARγ2, HSL and p-HSL. Compared with the HFD group, there was a significant decrease in the protein expression levels of PPARγ2 by 0.4% and 0.8% puerarin. The protein expression levels of p-AMPK, HSL and p-HSL were markedly increased in mice fed the 0.4% and 0.8% puerarin diets compared with the HFD group.

## Discussion

The aim of the present study was to investigate the anti-obesity effects of puerarin through its effects on body weight, IPAT weight, and serum biochemical parameters, as well as on activity, mRNA and protein levels of hepatic lipid metabolism enzymes in mice. Results strongly suggest that lipid levels in the serum and the liver were decreased by ≥0.4% puerarin, inhibiting body weight gain and IPAT weight in mice fed puerarin-containing diets. Moreover results showed that 0.4% and 0.8% puerarin increased the activities of CAT and HSL, and decreased the activity of FAS in the liver. Since the liver is known as an organ active for β-oxidation, stimulation of lipid metabolism may contribute to the suppression of hepatic and visceral fat accumulation [21]. FAS activity has been reported to be positively correlated with body fat [22]. The present study suggests that enhancement of β-oxidation and suppression of lipogenesis might be the major reasons for the reduction of fat accumulation and body weight increase in mice, supported by previous studies [23–25]. Therefore, a supplementation of ≥0.4% puerarin has a potential anti-obesity effect.

When fat cells increase in number and size, the ob gene starts to produce leptin, which is secreted into the circulation [26]. Serum leptin levels are directly proportional to adipose tissue weight [27]. Therefore, the reduction in leptin levels observed in the present study may be attributable, at least in part, to the decrease in IPAT weight in mice fed the puerarin diets.

AMPK is involved in a number of metabolic pathways and plays an important role in the regulation of cellular energy [28–30]. When activated by conditions of depleted energy such as hypoxia and glucose deprivation, AMPK suppresses ATP-consuming processes such as fatty acid synthesis and gluconeogenesis, and stimulates catabolic pathways that generate ATP such as β-oxidation, glycolysis and glucose uptake [28]. AMPK leads to a number of metabolic changes that could be potential targets in the treatment of metabolic disorders such as obesity, type 2 diabetes and metabolic syndrome [31]. In the present study, ≥0.4% puerarin significantly increased AMPK activity and p-AMPK protein levels, but no remarkable increases in mRNA and protein expression levels of AMPK were observed. These results might suggest that puerarin enhances AMPK activity via phosphorylation. Interestingly, the activities of AMPK, CAT and HSL were significantly increased by ≥0.4% puerarin, which may indicate that the enhanced activity of AMPK promoted the activity of CAT, and suppressed the activity of FAS, which are main contributors to the regulation of fat metabolism. HSL is the key enzyme of intracellular TG hydrolysis, and is a determinant of fatty acid mobilization in adipose and other tissues. AMPK activation significantly suppressed the basal and epinephrine-stimulated HSL activity and activity of adipose triglyceride lipase (ATGL) in adipose tissue [32]. HSL and ATGL, two enzymes critical for lipolysis in adipose tissues, have been found to play an important role in hepatic lipid homeostasis [33]. It was reported that HSL and ATGL account for more than 90% of TG hydrolase activity in mouse white adipose tissue [34]. The effects of TG hydrolase on white adipose tissue of ATGL^-/-^ mice is 80% lower than in wild-type mice, suggesting that ATGL is the key enzyme for TG hydrolysis in white adipose tissue, whereas HSL functions primarily as a diglyceride hydrolase [35]. In the present study, we investigated the effect of puerarin on the mRNA and protein expression of lipolysis enzymes in liver. The mRNA and protein expression levels of HSL were significantly up-regulated in mice fed 0.4% and 0.8% puerarin. Furthermore, p-HSL protein expression levels were also increased. These results suggest that puerarin increases HSL activity at both the mRNA and protein expression levels, and enhances its lipolysis effect via phosphorylation.

PPARγ is a regulator of adipocyte differentiation, and plays an important role in lipid metabolism, glucose homeostasis and cell proliferation [36]. PPARγ directly activates many genes involved in adipocyte lipid storage [37]. PPARγ is a potential physiologic sensor of lipid levels, linking fatty acids and other lipid-related molecules to glucose and lipid homeostasis [38]. PPARγ regulates the expression of adipogenic genes, and promotes the differentiation and proliferation of adipocytes, causing an increase in adipose tissue mass [39,40]. To the best of our knowledge, it has not been reported that puerarin can regulate the gene expression level of *PPARγ* in liver. In the present study, mRNA and protein levels of *PPARγ* were significantly down-regulated in mice fed 0.8% puerarin, indicating that puerarin enhanced fat metabolism and then contributed to energy expenditure, thus inhibited fat accumulation.

The present study is not without limitations. First, the puerarin extract used in the present study is the purest puerarin preparation commercially available, with a purity of 90% as determined by HPLC. However, the compounds in the remaining 10% are currently unidentified. They might be puerarin derivatives such as hydroxyl puerarin, methoxy puerarin, daidzin and daidzein. We cannot exclude the possibility that some of the biological effects might be due, at least in part, by these impurities, but it should be surprising since 90% of the preparation is pure puerarin. No glycolysis inhibitor was used in the serum samples, and the glucose levels could have suffered from glycolysis until samples were frozen at -80°C. Finally, no control group was fed puerarin along with a normal diet.

Taken together, the present study strongly suggests that puerarin affects the activities of hepatic lipid metabolism-related enzymes through regulation of mRNA and protein expression levels. The enhancement of lipolysis and suppression of fat synthesis were found to decrease lipid levels in serum and liver, and then decreased fat accumulation and body weight gain. The increased p-AMPK and p-HSL protein expression levels may promote lipolysis. These data suggest that puerarin might be used to prevent lifestyle-related diseases such as obesity, type 2 diabetes and metabolic syndrome.

## Supporting Information

S1 Table Composition of the diets. (DOCX)
