# Supplementary material for: Effects of Puerarin on Lipid Accumulation and Metabolism in High-Fat Diet-Fed Mice
Source: PLoS One. 2015 Mar 30;10(3):e0122925. doi: 10.1371/journal.pone.0122925 (PMC4378957; doi:10.1371/journal.pone.0122925)
Supplement: S1 Table — (DOCX) [file pone.0122925.s001.docx]

Table S1. Composition of the diets (g/kg)

| Ingredient | Control | HFD | HFD + 0.2% puerarin | HFD + 0.4% puerarin | HFD + 0.8% puerarin |
| --- | --- | --- | --- | --- | --- |
| Corn starch | 398 | 195 | 193 | 191 | 187 |
| Dextrin | 127 | 127 | 127 | 127 | 127 |
| Sucrose | 100 | 100 | 100 | 100 | 100 |
| Cellulose | 50 | 50 | 50 | 50 | 50 |
| Casein | 210 | 210 | 210 | 210 | 210 |
| Vitamin mix.^1^ | 10 | 10 | 10 | 10 | 10 |
| Minaeral mix.^2^ | 30 | 30 | 30 | 30 | 30 |
| DL-methionine | 3 | 3 | 3 | 3 | 3 |
| Choline bitartrate | 2 | 2 | 2 | 2 | 2 |
| Corn oil | 70 | 70 | 70 | 70 | 70 |
| Beef tallow | — | 200 | 200 | 200 | 200 |
| Cholesterol | — | 3 | 3 | 3 | 3 |
| Puerarin | — | — | 2 | 4 | 8 |
| Energy (kcal/kg) | 3982 | 4982 | 4974 | 4966 | 4950 |

^1,2^: Vitamin mixture and mineral mixture were prepared according to AIN-76TM.

Energy from sucrose, com starch, dextrin and casein were 4 kcal/g. Energy from corn oil and beef tallow were 9 kcal/g. Energy from cellulose was 0 kcal/g.
